# Ulcerative Colitis With Concomitant Serrated Polyposis Syndrome: A Case Report and Literature Review

**DOI:** 10.7759/cureus.14591

**Published:** 2021-04-20

**Authors:** Mahmoud M Mansour, Zachary D Smith, Yezaz Ghouri, Veysel Tahan

**Affiliations:** 1 Internal Medicine, University of Missouri School of Medicine, Columbia, USA; 2 Internal Medicine/Gastroenterology and Hepatology, University of Missouri School of Medicine, Columbia, USA

**Keywords:** inflammatory bowel disease, ulcerative colitis (uc), colorectal cancer (crc), sessile serrated adenoma, serrated polyp, serrated polyposis syndrome (sps)

## Abstract

Serrated polyposis syndrome (SPS) is a pre-cancerous condition associated with increased risk of developing colorectal cancer (CRC). Its role in inflammatory bowel disease (IBD)-associated CRC remains unknown. Despite the growing understanding and recognition of SPS, there is limited literature about its impact on the colon in individuals with IBD. Herein, we report a case of a 45-year-old female who was diagnosed with ulcerative colitis (UC) and SPS. We also reviewed the literature surrounding this association and highlighted the intricacies in managing this unique patient population. At present, there are no screening guidelines for CRC in SPS patients with IBD. However, given the potential synergistic risk for CRC, a close surveillance approach may be utilized. ﻿Tracking lifetime cumulative features of SPS and endoscopic clearance of adenomas and serrated polyps are the mainstays of management.

## Introduction

Patients with inflammatory bowel disease (IBD) i.e., ulcerative colitis (UC) and Crohn’s disease, have a higher risk of colorectal cancer (CRC) [1]. IBD-associated CRC, similar to sporadic CRC, develops through the dysplasia-carcinoma sequence [2]. The dysplastic lesions in IBD cytologically resemble sporadic tubular, villous, or serrated adenomatous phenotypes [3]. Serrated lesions are a pathologically diverse group divided into hyperplastic polyps, sessile serrated lesions with or without dysplasia, traditional serrated adenoma, and unclassified serrated adenomas [4].

Serrated polyposis syndrome (SPS), previously known as hyperplastic polyposis syndrome, is a relatively newly recognized condition characterized by the presence of serrated polyps that are multiple, large, and usually located in the proximal colon [5]. SPS is defined according to the recently updated 2019 World Health Organization (WHO) criteria. All subtypes of serrated lesions are included in the count, which is cumulative over subsequent endoscopies [6]. Current knowledge of IBD with concomitant SPS is limited, and clinical guidelines regarding management are lacking. This case report describes an interesting case of concurrent UC with SPS and a review of the literature surrounding this association. 

This article was previously presented as an abstract (Abstract: Smith Z, Yezaz G, Ibdah J, Tahan V. Ulcerative Colitis With Concomitant Serrated Polyposis Syndrome. American Journal of Gastroenterology; October 2019).

## Case presentation

SPS is defined according to the recently updated 2019 World Health Organization (WHO) criteria (Table 1).

**Table 1:** 2019 serrated polyposis syndrome (SPS) World Health Organization (WHO) criteria

2019 SPS WHO criteria	
Criteria I	≥5 serrated lesions proximal to the rectum, all being ≥5 mm in size, with at least two being ≥10 mm in size.
Criteria II	>20 serrated lesions of any size distributed throughout the large bowel, with at least five being proximal to the rectum.

**Figure 1:**
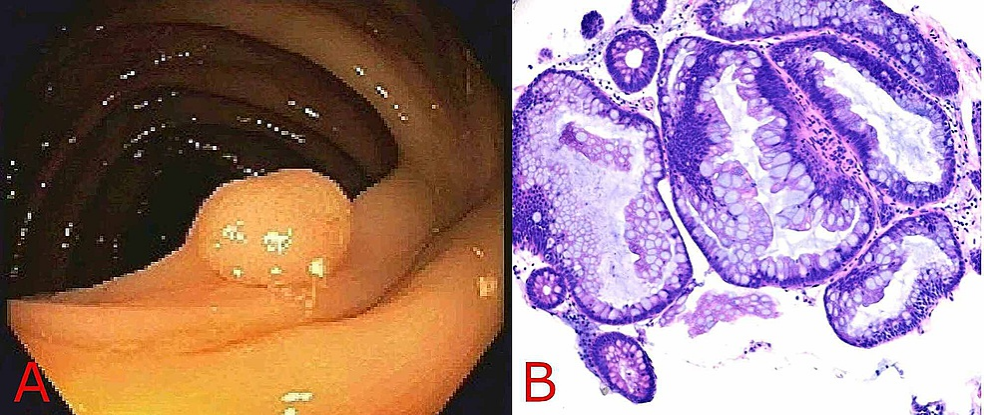
Ascending colon sessile serrated adenoma/polyp A: Endoscopic view showing ascending colon sessile serrated adenomatous polyp B: Hematoxylin-eosin stain of the resected specimen of sessile serrated adenoma without cytologic dysplasia showing the presence of serrated crypts and irregularly dilated and branching crypts.

**Figure 2:**
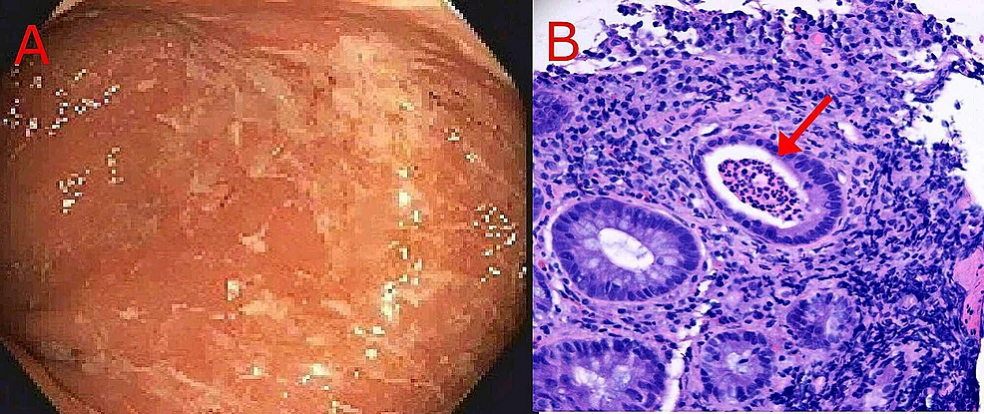
Proctitis; inflammation of the rectal mucosa A: Endoscopic view showing moderate rectal erythema and muco-purulent discharge. B: Hematoxylin-eosin stain of the rectal biopsy specimen showing active proctitis. A crypt abscess (arrow) is visible.

A 45-year-old female with no significant past medical history presented to the clinic with a two-month history of change in bowel habits, bloating, and occasional hematochezia. She denied a family history of CRC or IBD. Colonoscopy revealed three flat polyps (Paris IIb) measuring 5 mm, 7 mm, and 8 mm in size at the cecal base and two flat polyps (Paris IIb) each 11 mm in size in the ascending colon (Figure 1A). All polyps were removed with a snare and found to be of sessile serrated type on histopathologic examination, thereby meeting the first WHO criteria for SPS (Figure 1B). The patient was also found to have a 20 cm segment of inflammation in the rectum, whose biopsies showed diffuse active proctitis with severe inflammatory activity, suggestive of ulcerative proctitis (Figures 2A-2B). The patient was treated with mesalamine 1000 mg rectal suppository daily with a plan for repeating colonoscopy in six months. 

## Discussion

Serrated polyps are the presumptive precursor for the development of up to one-third of sporadic CRC [7]. In contrast to the traditional adenoma-carcinoma pathway that usually involves APC mutations, the serrated pathway commonly has mutations in BRAF proto-oncogenes and develops excessive methylation of the CpG promoter regions of mismatch repair genes (such as MLH-1), resulting in microsatellite instability [8,9]. This molecular background that hastens carcinogenesis, in addition to the indistinct and flat nature of serrated polyps, could explain serrated polyps’ implication in “interval cancers”, which are cancers that are diagnosed before the next recommended colonoscopy [10].

Three retrospective studies have examined serrated polyps in IBD [11-13]. Two of these studies suggested that there may be an additional risk of neoplasia in serrated lesions associated with IBD when compared to the general population, suggesting the need for closer surveillance [13]. However, conclusions are difficult to be drawn, as these studies had significant variability in patient selection, pathologic terminology, and outcomes recorded. Additionally, the small sample size limits further sub-group analysis as the majority of patients had hyperplastic polyps, which carry the least neoplastic potential. To date, there are no specific clinical recommendations for serrated lesions in IBD patients.

Historically, SPS was thought to be a rare condition; however, in the last decade, reporting of SPS has been increasing with greater clinical and pathological awareness, better endoscopic diagnostic accuracy, and systematic tracking of polyps [14]. In fact, SPS has become the most common polyposis syndrome currently known, with a prevalence of up to 0.4% in average risk colonoscopies [15] and 0.8% in colonoscopies following a positive fecal immunochemical testing [16]. Furthermore, it is currently well recognized that SPS is a significant contributor to CRC development, with CRC occurring in 15.8%-29.3% of patients with SPS [17,18]. 

SPS is a heterogeneous syndrome likely influenced by a variety of environmental factors like smoking and genetic modifiers [19]. Although, some genetic abnormalities have been linked to SPS, such as germline RNF43 mutation, the majority of patients with the syndrome do not have an identifiable germline mutation [20].

When it comes to the IBD population, SPS is rarely reported in the literature. In addition to our patient, there are ten other IBD cases associated with SPS described across three case series [21-23]. All but two of the reported patients had a long-standing history of IBD for over ten years. Seven patients had UC, and four patients had CD. In four patients, the WHO criteria for SPS was met after one or more colonoscopies were performed over a period of 1-3 years, highlighting the importance of systematic tracking of polyps. A cohort study has shown that up to 45% of SPS cases are not diagnosed at first colonoscopy [24].

In the more recently reported cases, surveillance colonoscopies in IBD patients were performed using chromoendoscopy (CE), which appears to have improved the detection yield of polyps [22,23]. In fact, in one of the patients, white light endoscopies failed to detect any significant lesions [23]. These observations underline the utility of CE, in which dye is applied to the colonic mucosa to improve the detection of dysplasia. In a meta-analysis involving six studies with IBD patients, CE improved the detection rate of dysplastic lesions by 44% [25].

Multiple cohort studies have discussed endoscopic surveillance regimens in SPS patients [14,26]. While some guidelines recommend strict annual surveillance [27], others recommend a more personalized approach, based on the variable risk factors for developing CRC, in an attempt to reduce colonoscopy burden [14,28]. However, it remains unclear if SPS carries a synergetic effect on the risk of CRC when combined with IBD. Currently, there are no recommendations for screening patients with concomitant IBD and SPS, but given the potential additive risk for CRC, a close surveillance approach was recommended for the reported patients, with repeat screening intervals between three and twelve months.

## Conclusions

This case highlights the complexity of managing patients with concurrent IBD and SPS. With the increasing recognition and understanding of SPS, additional studies are needed to further elucidate the natural history and define surveillance strategies for this syndrome in patients with IBD.

## References

[1] Biancone L, Armuzzi A, Scribano ML (2020). Cancer risk in inflammatory bowel disease: a 6-year prospective multicenter nested case-control IG-IBD study. Inflamm Bowel Dis.

[2] Magro F, Langner C, Driessen A (2013). European consensus on the histopathology of inflammatory bowel disease. J Crohns Colitis.

[3] (2017). Gastrointestinal pathology. Mod Pathol.

[4] Nagtegaal ID, Odze RD, Klimstra D (2020). The 2019 WHO classification of tumours of the digestive system. Histopathology.

[5] Renaut AJ, Douglas PR, Newstead GL (2002). Hyperplastic polyposis of the colon and rectum. Colorectal Dis.

[6] Rosty C, Brosens L, Dekker E, Nagtegaal I (2019). Serrated polyposis. WHO Classification of Tumours of the Digestive System, 5th Edition.

[7] Makkar R, Pai RK, Burke CA (2012). Sessile serrated polyps: cancer risk and appropriate surveillance. Cleve Clin J Med.

[8] Borowsky J, Dumenil T, Bettington M (2018). The role of APC in WNT pathway activation in serrated neoplasia. Mod Pathol.

[9] Minoo P, Moyer MP, Jass JR (2007). Role of BRAF-V600E in the serrated pathway of colorectal tumourigenesis. J Pathol.

[10] Burgess NG, Tutticci NJ, Pellise M, Bourke MJ (2014). Sessile serrated adenomas/polyps with cytologic dysplasia: a triple threat for interval cancer. Gastrointest Endosc.

[11] Jackson WE, Achkar JP, Macaron C (2016). The significance of sessile serrated polyps in inflammatory bowel disease. Inflamm Bowel Dis.

[12] Shen J, Gibson JA, Schulte S (2015). Clinical, pathologic, and outcome study of hyperplastic and sessile serrated polyps in inflammatory bowel disease. Hum Pathol.

[13] Ko HM, Harpaz N, McBride RB (2015). Serrated colorectal polyps in inflammatory bowel disease. Mod Pathol.

[14] Bleijenberg AG, IJspeert JEG, van Herwaarden YJ (2020). Personalised surveillance for serrated polyposis syndrome: results from a prospective 5-year international cohort study. Gut.

[15] IJspeert JEG, Bevan R, Senore C (2017). Detection rate of serrated polyps and serrated polyposis syndrome in colorectal cancer screening cohorts: a European overview. Gut.

[16] Moreira L, Pellisé M, Carballal S (2013). High prevalence of serrated polyposis syndrome in FIT-based colorectal cancer screening programmes. Gut.

[17] Carballal S, Rodríguez-Alcalde D, Moreira L (2016). Colorectal cancer risk factors in patients with serrated polyposis syndrome: a large multicentre study. Gut.

[18] IJspeert JEG, Rana SAQ, Atkinson NSS (2017). Clinical risk factors of colorectal cancer in patients with serrated polyposis syndrome: a multicentre cohort analysis. Gut.

[19] Walker RG, Landmann JK, Hewett DG (2010). Hyperplastic polyposis syndrome is associated with cigarette smoking, which may be a modifiable risk factor. Am J Gastroenterol.

[20] Quintana I, Mejías-Luque R, Terradas M (2018). Evidence suggests that germline RNF43 mutations are a rare cause of serrated polyposis. Gut.

[21] Srivastava A, Redston M, Farraye FA, Yantiss RK, Odze RD (2008). Hyperplastic/serrated polyposis in inflammatory bowel disease: a case series of a previously undescribed entity. Am J Surg Pathol.

[22] Feuerstein JD, Flier SN, Yee EU, Pleskow D, Cheifetz AS (2014). A rare case series of concomitant inflammatory bowel disease, sporadic adenomas, and serrated polyposis syndrome. J Crohns Colitis.

[23] Castro J, Cuatrecasas M, Balaguer F, Ricart E, Pellisé M (2017). Serrated polyposis syndrome associated with long-standing inflammatory bowel disease. Rev Esp Enferm Dig.

[24] Vemulapalli KC, Rex DK (2012). Failure to recognize serrated polyposis syndrome in a cohort with large sessile colorectal polyps. Gastrointest Endosc.

[25] Subramanian V, Mannath J, Ragunath K, Hawkey CJ (2011). Meta-analysis: the diagnostic yield of chromoendoscopy for detecting dysplasia in patients with colonic inflammatory bowel disease. Aliment Pharmacol Ther.

[26] IJspeert JE, Rana SA, Atkinson NS (2017). Clinical risk factors of colorectal cancer in patients with serrated polyposis syndrome: a multicentre cohort analysis. Gut.

[27] Lieberman DA, Rex DK, Winawer SJ, Giardiello FM, Johnson DA, Levin TR (2012). Guidelines for colonoscopy surveillance after screening and polypectomy: a consensus update by the US Multi-Society Task Force on Colorectal Cancer. Gastroenterology.

[28] Dekker E, Bleijenberg A, Balaguer F (2020). Update on the World Health Organization criteria for diagnosis of serrated polyposis syndrome. Gastroenterology.

